# Recent Advances of Actinomycetes

**DOI:** 10.3390/biom11020134

**Published:** 2021-01-21

**Authors:** Eung-Soo Kim

**Affiliations:** Department of Biological Engineering, Inha University, Incheon 22212, Korea; eungsoo@inha.ac.kr; Tel.: +82-32-860-8318

The discovery and development of actinomycete secondary metabolites (ASMs) have played pivotal roles in the fields of human medicine and its related biotechnology sectors over the past several decades. The postgenomic era has enabled the development of a genome mining approach to isolate and characterize previously unsuspected ASM biosynthetic gene clusters (BGCs) hidden in the actinomycete genomes. Subsequently, BGC awakening techniques and biosynthetic mechanism studies have been pursued to maximize the biotechnological potential of actinomycetes. Moreover, chemical and synthetic biology approaches have allowed for higher production yields of novel and valuable ASMs, which could complement traditional culture-based approaches for chasing ASMs. This Special Issue focuses on recent advances in the study of actinomycetes, especially updated overviews of some significant ASMs and related technologies, including genome mining, BGC refactoring, and cell factory design. This Special Issue includes 11 original research papers and four review articles by renowned experts in the field, providing interested readers with specific examples of recent progress in the study of actinomycetes and ASMs.

The study by E. Kim et al. [1] characterizes the BGC of the cyclic depsipeptide ohmyungsamycin (OMS) from the marine-derived *Streptomyces* sp. SNJ042. Biosynthetic gene inactivation and analysis of the accumulated products provided the OMS biosynthetic mechanism as well as generation of OMS derivatives. Interestingly, some of the OMS derivatives exhibited significantly improved antituberculosis activities with decreased cytotoxicities compared to wild-type OMS A, implying that the characterization and engineering of OMS BGC could be an efficient way to obtain OMS structural diversity.

The study by X. Wang et al. [2] reveals that the antitumor agent named ansamitocin P-3 (AP-3) binds to cell division protein FtsZ in *Actinosynnema pretiosum*, a rare actinomycete. FtsZ, a β-tubulin analog present in bacteria, was discovered to be the AP-3 target through structural comparison and an Surface Plasmon Resonance (SPR) biosensor assay. Overexpression of the gene coding for FtsZ in *A. pretiosum* stimulated the resistance to AP-3 as well as AP-3 yield, suggesting that the FtsZ is a newly identified bacterial target of AP-3 and its resistance improvement is an effective strategy to enhance AP-3 production.

The study by C. Maruyama et al. [3] focuses on the biosynthetic enzymes and their mechanisms involved in the biosynthesis of 1-amino-2-methylcyclopropanecarboxylic acid (MeACC). The authors identified a BGC containing bacterial MeACC synthase genes. In vitro analysis using their recombinant enzymes further revealed that the ACC structure of MeACC is derived from the L-methionine moiety of S-adenosyl-L-methionine (SAM) and *C*-methylation of SAM occurs prior to cyclopropanation in MeACC biosynthesis.

Y. Wang et al. [4] rationalized the nonenzymatic coupling mechanism responsible for specific regio- and enantioselectivity of fluostatin conjugation by density functional theory calculations. Fluostatins belong to the benzofluorene-containing aromatic polyketides in the atypical angucycline family, which conjugate into dimeric and even trimeric compounds postbiosynthesis. The authors confirmed that the π–π stacking interactions within the fluostatin 6-5-6 aromatic ring system play a critical role in the stereoselectivity of the nonenzymatic fluostatin conjugation.

The study by Y. Wu et al. [5] explores the subtilisin-involved morphology engineering for improvement of an antitumor agent ansamitocin P-3 (AP-3) in* A. pretiosum* ATCC 31280. The authors identified a subtilisin-like serine peptidase encoding gene *APASM_4178*, which is responsible for the mycelial fragmentation through comparative transcriptomic analysis. *APASM_4178* deletion was found to result in increased biomass and improved AP-3 yield, implying that modulation of a subtilisin-like serine peptidase gene expression could be an effective strategy for morphology engineering and antibiotic yield improvement in actinomycetes.

The study by W. Kim et al. [6] explores complete genome sequences of three *S. venezuelae* strains (ATCC 10712, ATCC 10595, and ATCC 21113) harboring chloramphenicol and jadomycin BGCs. The high-quality genome sequences exhibited that the three strains share more than 85% of total genes, including most of the SM BGCs. Since these *S. venezuelae* strains were observed to produce different levels of SMs with the strain-specific regulation mechanism, genome sequences of closely related strains would serve as useful resources for understanding and optimization of *Streptomyces* SM biosynthesis.

The study by Y.H. Ban et al. [7] explores the development of new aminoglycoside (AG) antibiotics to overcome the resistance mechanism of AG-modifying enzymes (AMEs) in AG-resistant pathogens. The authors enzymatically synthesized new 6′-*N*-acylated isepamicin (ISP) analogs, which showed activities against the ISP-resistant Gram-negative bacteria and reduced toxicity compared to ISP in vitro. These results suggest that the regioselective enzymatic modification of AG scaffolds could be an efficient approach for the development of novel and improved AG antibiotics.

The study by X. Shen et al. [8] explores the isolation and structural elucidation of a group of naphthoquinone-containing compounds from *Streptomyces* sp. B9173. Seven flaviogeranin congeners or intermediates, including three new compounds, were confirmed to be derived from the common naphthoquinone backbone and subsequent oxidation, methylation, prenylation, and amino group incorporation. The chemical elucidation and proposed biosynthetic pathway extend the structural diversity of naphthoquinone-based meroterpenoids and provide new insights into their biosynthesis.

A. Teshima et al. [9] investigated the role of a P450 monooxygenase gene *srrO* and butenolide-type signaling molecule SRB synthase gene *srrX* from *S. rochei* 7434AN4. SRB1 is known to control two structurally unrelated polyketide antibiotics, namely lankacidin and lankamycin, in *S. rochei* 7434AN4. The authors performed bioconversion of the synthetic 6′-deoxo-SRB1 in the *S. lividans* recombinant carrying SrrO-expression plasmids. Substrate 6′-deoxo-SRB1 was converted through 6′-deoxo-6′-hydroxy-SRB1 to SRB1 in a time-dependent manner, implying that SrrO catalyzes the C-6′ oxidation at the final step in SRB biosynthesis. 

The study by F. Ye et al. [10] explores the production of 8-deoxy-rifamycin derivatives from *A. mediterranei* S699. Although proansamycin X, a hypothetical macrocyclic precursor in the rifamycin biosynthesis, had never been identified, bioinformatics analysis indicated that a putative NADH-dependent dehydrogenase gene named *rifT* might be a candidate target responsible for the dehydrogenation of proansamycin X. The authors generated an *A. mediterranei* S699 *ΔrifT* mutant strain, which successfully produced 11 8-deoxy-rifamycin derivatives and seven known analogs.

H. AbdElgawad et al. [11] isolated several bioactive *Streptomyces* species from the soil rhizosphere of legume crops. The authors claim that these isolates increase soil nutrients and organic matter content and improve soil microbial populations, implying that the usage of actinomycetes as biofertilizers could provide sustainable food production and achieve biosafety.

The review article by Y. Zhao et al. [12] summarizes the challenges and recent advances in *Streptomyces* genome editing tools, including the clustered regularly interspaced short palindromic repeats (CRISPR)/CRISPR-associated protein (Cas) systems. Additionally, the authors discuss the future research focus, especially the development of endogenous CRISPR/Cas-based genome editing technologies in *Streptomyces *species.

The review article by C. Sun et al. [13] reveals the mechanisms and functions of protein post-translational modification (PTM) identified in actinomycetes, focusing on phosphorylation, acylation, and protein degradation in an attempt to summarize the recent progress of research on PTMs and their important role in bacterial cellular processes.

The review article by M. Kobayashi et al. [14] describes the carbazole biosynthetic mechanism, which includes a key step in the enzymatic formation of a tricyclic carbazole skeleton, followed by modifications such as prenylation and hydroxylation in the skeleton. 

The review article by S. Choi et al. [15] summarizes recent progress on microbial muonic acid (MA) production, including dehydroshikimate (DHS) pathway engineering and the heterologous expression of foreign pathways for DHS-to-MA conversion in *Corynebacterium glutamicum*. The authors suggest that microbial MA production represents a feasible way to complement various petrochemical-based chemical processes and will continue to play a vital role in the field of biorefineries.

This Special Issue describes important recent findings and key reviews on actinomycetes and related ASMs. Understanding and applying the ASMs should continue to play a critical role in the field of biotechnology, including in the screening of novel bioactive compounds.
